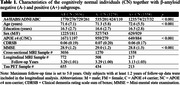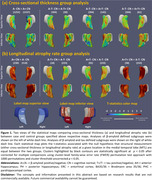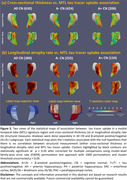# Spatial Pattern of Medial Temporal Lobe Cross‐Sectional and Longitudinal Structural Change in Cognitively Normal Individuals

**DOI:** 10.1002/alz.093969

**Published:** 2025-01-09

**Authors:** Long Xie, Sandhitsu R. Das, Yue Li, Laura E.M. Wisse, Emily McGrew, Xueying Lyu, Michael DiCalogero, Ujashi Shah, Ademola Ilesanmi, Amanda E Denning, Christopher D. Brown, Jesse S Cohen, Lasya P Sreepada, Mengjin Dong, Niyousha Sadeghpour, Pulkit Khandelwal, Ranjit Ittyerah, Sadhana Ravikumar, Shokufeh Sadaghiani, Stanislau Hrybouski, Robin de Flores, Eli Gibson, Paul A. Yushkevich, David A Wolk

**Affiliations:** ^1^ Siemens Healthineers, Princeton, NJ USA; ^2^ Penn Image Computing and Science Laboratory (PICSL), University of Pennsylvania, Philadelphia, PA USA; ^3^ University of Pennsylvania, Philadelphia, PA USA; ^4^ Department of Clinical Sciences Lund, Lund University, Lund, Lund Sweden; ^5^ Perelman School of Medicine, University of Pennsylvania, Philadelphia, PA USA; ^6^ Department of Neurology, University of Pennsylvania, Philadelphia, PA USA; ^7^ Normandie Univ, UNICAEN, INSERM, U1237, PhIND Physiopathology and Imaging of Neurological Disorders, NeuroPresage Team, GIP Cyceron, Caen France; ^8^ Siemens Heathineers, Princeton, NJ USA

## Abstract

**Background:**

The medial temporal lobe's (MTL) early involvement in tau pathology makes it a key focus in the development of preclinical Alzheimer’s disease (AD) biomarkers. ROI analyses in prior studies reported significant MTL structural differences in cognitively normal individuals with and without ß‐amyloid (A+/‐CN). Pointwise analysis, offering spatial information of early neurodegeneration, has potential to pinpoint “signature regions” of pathological change, but has been underexplored in the MTL. This study employs a specialized pointwise analysis pipeline to examine the spatial pattern of MTL structural change in subgroups dichotomized by both ß‐amyloid and tau status in a large cohort of CN individuals.

**Methods:**

A dataset of 3036 CN (A‐/A+: 1270/1558, Table 1) individuals from ADNI, HABS, A4 and ABC were analyzed. We extracted MTL regional thickness maps from MRI using tailored pipelines, ASHS‐T1 and CRASHS. For participants with prospective longitudinal MRI (five years follow‐up), regional maps of longitudinal atrophy rate were extracted using SkelDBM. Subjects with cross‐sectional tau PET available (N=563) were further divided into A and T subgroups by tracer uptake. General linear modeling was performed on each surface point to investigate cross‐sectional and longitudinal MTL structural group differences (detailed in Figure 1) and their correlation with MTL tau burden in All/A+/A‐ CN. Age and sex were covariates and cluster‐level multiple comparison correction was performed.

**Results:**

A+CN demonstrated a significantly faster atrophy rate than A‐CN across the whole MTL, primarily driven by A+T+CN individuals (Figure 1‐b). Notably, A‐T+CN showed significantly faster atrophy rate in the entorhinal cortex (ERC) and Brodmann area 35 (BA35), the earliest sites of tau pathology (Figure 1‐b, second column). Figure 2‐b displays an MTL‐wise significant correlation between atrophy rate and tau in All/A+/A‐ CN. In both analyses, cross‐sectional effects are consistently weaker than longitudinal ones, but have some significant clusters in ERC and BA35.

**Conclusions:**

Pointwise analysis revealed extensive tau‐associated accelerated neurodegeneration in the MTL in preclinical AD. Furthermore, accelerated atrophy was observed in early Braak regions in A‐CN with evidence of tau pathology, potentially driven by primary age‐related tauopathy (PART). These pointwise longitudinal MTL measures provide sensitive measures that may allow for disease monitoring in preclinical AD.